# Comparison of the Specificities of IgG, IgG-Subclass, IgA and IgM Reactivities in African and European HIV-Infected Individuals with an HIV-1 Clade C Proteome-Based Array

**DOI:** 10.1371/journal.pone.0117204

**Published:** 2015-02-06

**Authors:** Daniela Gallerano, Portia Ndlovu, Ian Makupe, Margarete Focke-Tejkl, Kerstin Fauland, Eva Wollmann, Elisabeth Puchhammer-Stöckl, Walter Keller, Elopy Sibanda, Rudolf Valenta

**Affiliations:** 1 Division of Immunopathology, Department of Pathophysiology and Allergy Research, Medical University of Vienna, Vienna, Austria; 2 Gamma City Laboratory, Harare, Zimbabwe; 3 Institute of Molecular Biosciences—Structural Biology, Karl Franzens University, Graz, Austria; 4 Department of Virology, Medical University of Vienna, Vienna, Austria; 5 Asthma, Allergy and Immune Dysfunction Clinic, Parirenyatwa University Teaching Hospital, Harare, Zimbabwe; University of Massachusetts Medical Center, UNITED STATES

## Abstract

A comprehensive set of recombinant proteins and peptides of the proteome of HIV-1 clade C was prepared and purified and used to measure IgG, IgG-subclass, IgA and IgM responses in HIV-infected patients from Sub-Saharan Africa, where clade C is predominant. As a comparison group, HIV-infected patients from Europe were tested. African and European patients showed an almost identical antibody reactivity profile in terms of epitope specificity and involvement of IgG, IgG subclass, IgA and IgM responses. A V3-peptide of gp120 was identified as major epitope recognized by IgG_1_>IgG_2_ = IgG_4_>IgG_3_, IgA>IgM antibodies and a C-terminal peptide represented another major peptide epitope for the four IgG subclasses. By contrast, gp41-derived-peptides were mainly recognized by IgG_1_ but not by the other IgG subclasses, IgA or IgM. Among the non-surface proteins, protease, reverse transcriptase+RNAseH, integrase, as well as the capsid and matrix proteins were the most frequently and strongly recognized antigens which showed broad IgG subclass and IgA reactivity. Specificities and magnitudes of antibody responses in African patients were stable during disease and antiretroviral treatment, and persisted despite severe T cell loss. Using a comprehensive panel of gp120, gp41 peptides and recombinant non-surface proteins of HIV-1 clade C we found an almost identical antibody recognition profile in African and European patients regarding epitopes and involved IgG-sublass, IgA- and IgM-responses. Immune recognition of gp120 peptides and non-surface proteins involved all four IgG subclasses and was indicative of a mixed Th1/Th2 immune response. The HIV-1 clade C proteome-based test allowed diagnosis and monitoring of antibody responses in the course of HIV-infections and assessment of isotype and subclass responses.

## Introduction

Since the first reports of patients suffering from severe immunodeficiency in 1981 [1, 2] and the consecutive identification of human immunodeficiency virus type 1 (HIV-1) as a causative agent for the underlying destruction of the immune system [3], millions of patients worldwide have been affected by HIV-1 infections [4]. HIV-1 belongs to the family of *Retroviridae* and to the species of primate lentiviruses that affect hematopoietic cells [5]. HIV-1 infection is associated with progressive CD4 T cell loss and immune dysfunction caused by several mechanisms such as chronic T cell activation, chronic antigen presentation and dysregulated immune cell homeostasis, which can lead to acquired immunodeficiency syndrome (AIDS) [6]. One direct cause of CD4+ T cell loss is that HIV-1 infects CD4+ T cells by using CD4 as entry-receptor [7]. Chemokine receptors CCR5 and CXCR4 can function as co-receptors for HIV and contribute to tropic and biological properties of HIV isolates [8]. Surface envelope glycoprotein (gp120) and transmembrane envelope glycoprotein (gp41) are the structures involved in infection of host cells [9]. Gp120 and gp41 are highly glycosylated proteins that form trimeric structures that appear in form of spikes on the virus surface [10, 11]. Attempts to develop specific immune intervention strategies such as vaccines or neutralizing therapeutic antibodies have particularly focused on the HIV envelope proteins gp120 and gp41 [12–14]. The extraordinary genetic diversity of existing HIV subtypes or clades resulting in a broad antigenic diversity of the envelope has been postulated as another obstacle for the development of broadly effective immune intervention strategies [12]. In fact, HIV-1 occurs in several genetic subtypes which can be classified into four groups: M (main), O (outlier), N (non-M, non-O) and group P, of which group M, consisting of at least 9 distinct clades, includes 95% of the global virus isolates [15, 16]. Among HIV-1 subtypes, clade C, A and B are the most prevalent, causing 48%, 12% and 11% of worldwide infections, respectively [17]. In particular, HIV-1 clade C has become the most dominant subtype particularly in Southern Africa and India [18], whereas clade B viruses are the most prevalent in Europe and North America [17, 19].

Several studies have investigated the epitope specificity of the polyclonal antibody responses of HIV-infected patients against gp120 or gp41 for certain strains [20–24]. One recent study has analyzed polyclonal antibody responses of vaccinated persons and infected persons with peptides spanning gp120 and gp41 [25], but so far the full spectrum of antibody response in terms of antibody classes and subclasses against an almost complete proteome of a certain strain represented by peptides spanning the envelope and recombinant viral proteins has not been compared in HIV-infected patients from different continents and during the course of disease.

In this study we used recombinant protein expression and synthetic-peptide-chemistry to assemble an almost complete proteome of HIV-1 clade C as an array of antigens and epitopes to compare specificity, type (isotype, subclass) and magnitude of antibody responses in a population of HIV-infected patients from Sub-Saharan Africa and compared the results to a cohort of HIV-infected patients from Europe, where clade B is prevalent. Furthermore, the protein/peptide array was used to monitor antibody responses in patients over the natural course of disease and during treatment.

## Materials and Methods

### Synthesis of HIV-1 clade C-derived peptides

Overlapping peptides covering the complete amino acid sequences of gp120 and gp41 from South African HIV-1 clade C reference strain (isolate ZA.04.04ZASK146, Los Alamos HIV sequence database accession no. AY772699) were produced by solid-phase-synthesis (CEM-Liberty, Matthews, NC, USA; Applied Biosystems, Life technologies, Carlsbad, CA, USA) (S1 Table). The synthesis was performed with the 9-fluorenyl-methoxy-carbonyl (Fmoc)-method, using PEG-PS preloaded resins [26]. Synthesized peptides were washed with dichloromethane, cleaved from the resins in a mixture of 19ml trifluoroacetic acid, 500ul silane and 500ul H_2_O and precipitated into pre-chilled *tert*-butylmethylether. Peptides were separated from by-products by reverse-phase HPLC in a 10–70% acetonitrile gradient on a Jupiter 4μm Proteo 90Å, LC column (Phenomenex, Torrance, CA, USA) with an UltiMate 3000 Pump (Dionex, Sunnyvale, CA, USA) to a purity >90%. Their identity was verified by mass spectrometry (Microflex MALDI-TOF, Bruker, Billerica, MA, USA). Peptides 120/3 and 41/10 were synthesized by pi-Chem (Graz, Austria). The peptides’ chemical features, were analyzed by ProtParam, on the Expasy proteomics server and were considered in the optimization of the solubilization conditions. Highly hydrophobic peptides were solubilized in 2–10% v/v di-methylformamide, 2–5% v/v di-methylsulfoxide or 5–10% v/v acetonitrile, while reducing agents (e.g. 1mM di-thio-erythritol) were added to cysteine-rich-peptides and 2–6mM NaOH to acidic peptides insoluble in water.

### Expression and purification of recombinant HIV-1 clade C proteins

HIV-1 clade C recombinant matrix, capsid, nucleocapsid proteins as well as accessory NEF, TAT and VIF and the *pol*-derived protease, reverse transcriptase+RNAseH and integrase were expressed in *Escherichia coli* (*E*.*coli)*. The cDNA sequences of the structural and accessory proteins as well as of the protease were from the South African HIV-1 clade C reference strain (isolate ZA.04.04ZASK146, Los Alamos HIV sequence database accession no. AY772699). Sequences of the reverse transcriptase-RNAseH and integrase were from the Ethiopian HIV-1 clade C isolate ET.86.ETH2220 (Los Alamos HIV sequence database accession no. U46016) because no complete sequences were available from isolate ZA.04.04ZASK146. Analysis of sequences available in the HIV sequence database (hiv.lanl.gov) showed that sequences isolated in Zimbabwe from where the analyzed sera were derived, were highly similar to the South African reference strain (Clustal W identity scores: Env 79%, Gag 90%, Pol 93%, Nef 88%) and to the Ethiopian reference strain (Pol 92%) which we used in our clade C array.

cDNAs coding for the proteins containing a C-terminal hexa-histidine tag were codon-optimized for bacterial expression and cloned into *EcoR* I and *Nde* I sites of plasmid pET17b (ATG:biosynthetics, Merzhausen, Germany). Transformed *E*.*coli* BL21(DE3) cells were grown to an *OD*_600_ = 0.4–0.6 in LB medium supplemented with 100mg/l ampicillin and expression was induced with 0.5–1.0mM isopropyl-β-thiogalactopyranoside. The time-points of maximal expression of the recombinant proteins were established by time-course analyses performed as described previously [27].

Matrix, capsid and nucleocapsid proteins were harvested after 4h, 4h and overnight expression, respectively, and purified under native conditions by lysis of cells in 50mM NaH_2_PO_4_, 300mM NaCl, 10mM Imidazole pH8.0, PMSF (1ul/ml), Lysozyme (1mg/ml) by sequential cycles of freezing and thawing. DNA was cleaved by treatment with 5ug/ml DNaseI (20min at room temperature); and, after removal of cellular debris (by centrifugation at 15,000xg, 20min, 4°C), the clear lysate was incubated with Nickel-agarose (QIAGEN, Hilden, Germany) 2–4h at 4°C. Elution of the His-tagged proteins was performed with an imidazole gradient (20, 50, 100, 250mM Imidazole) in 50mM NaH_2_PO4, 300mM NaCl pH 8.0.

Optimal expression of NEF and TAT was achieved at 4h post-induction and at 30min for protease, which was toxic for *E*. *coli*. The three proteins were purified under denaturing conditions, by lysing cells in 8M Urea or 6M Guanidinium chloride buffer for at least 2h or overnight at room temperature. Cellular debris were removed by centrifugation at 15,000xg (20min, 4°C), the clear lysates were incubated with Nickel-agarose (QIAGEN) for 2–4h at room temperature or overnight at 4°C, and a pH gradient (pH 6.3, 5.6, 4.5) was used to elute the recombinant proteins in 8M Urea, 100mM NaH_2_PO_4_, 10mM Tris.

For purification of recombinant integrase, cells were harvested after 4h expression and lysed under native conditions. Since after centrifugation (15,000xg, 20min, 4°C) the protein was found in the insoluble fraction, this fraction was re-suspended in 8M Urea, 100mM NaH_2_PO_4_, 10mM Tris, pH8.0 and incubated at room temperature overnight. After removal of cellular debris by a second step of centrifugation at 15,000xg, 20min, 4°C, recombinant integrase was purified under denaturing conditions in 8M Urea. Purification of VIF and reverse transcriptase+RNAseH, after 4h expression, was achieved by using the previously described inclusion body preparation protocol [27]. Removal of urea and refolding were done by sequential dialysis steps. HIV-1 clade C derived recombinant gp120 (rgp120) expressed in 293 cells, was purchased from Sino Biological (Beijing, People’s Republic of China) [28]. The identity of the proteins was verified by SDS-PAGE and Coomassie staining, as well as by Westernblot (i.e. with mouse a-His-tag antibody, Dianova, Hamburg, Germany, and alkaline-phosphatase-coupled rabbit anti-mIgG, BD, Franklin Lakes, NJ, USA) and by mass spectrometry (Microflex MALDI-TOF, Bruker). Their secondary structures and thermal stabilities were analyzed by circular dichroism spectroscopy on a Jasko J-810 spectropolarimeter (Japan Spectroscopic, Tokyo, Japan), as previously described [27]. The biochemical features of the recombinant HIV-1 clade C proteins were analyzed with ProtParam (Expasy).

### Demographic, clinical and immunological characterization of study subjects

Sera from 15 African HIV-infected patients and from two asymptomatic, HIV-exposed subjects were from the Asthma, Allergy and Immune Dysfunction Clinic, Harare, Zimbabwe. For comparison, sera from 15 European HIV-infected patients and from 10 uninfected individuals were obtained from the Department of Virology of the Medical University of Vienna. Detailed demographic, clinical and serological data of the African subjects are summarized in S2 Table. Each of the subjects was tested for HIV by Line-Immuno-Assay (InnoLIA, Innogenetics, Gent, Belgium) and the asymptomatic subjects were additionally tested with the Abbott Murex HIV Ag/Ab combination (Abbott, North Chicago, IL, USA). Written informed consent was obtained from patients from Zimbabwe to use their anonymized serum samples for retrospective analyses. Sera from Austrian subjects were residual samples from a diagnostic laboratory; therefore no consent could be obtained for the anonymized analysis of the latter samples. However, the analysis of serum samples from both study groups and the consent procedure were approved by the ethics committee of the Medical University of Vienna, Austria (EK592/2010). All data were analyzed and reported anonymously.

### Determination of HIV-1 clade C protein and peptide-specific IgG, IgA and IgM

ELISA plates (Nunc Maxisorp, Thermo-Fisher Scientific, Waltham, MA, USA) were coated overnight at 4°C with peptides, recombinant proteins and human serum albumin (negative control) (Behring, King of Prussia, PA, USA) diluted in 100mM sodium-bicarbonate-buffer pH9.6 (2ug/ml). After washing in PBS, 0.05% v/v Tween20 (PBS/T) and blocking in 2% w/v BSA, PBS/T (4h, room temperature), plates were incubated with sera (1:200, 0.5% w/v BSA, PBS/T) overnight at 4°C. Bound antibodies were detected with mouse anti-human IgG_1_, IgG_2_, IgG_4_, IgA, IgM (1:1000, BD) or mouse anti-human IgG_3_ (1:5000, Sigma Aldrich, St. Louis, MO, USA) 2h at room temperature, followed by horseradish-peroxidase (HRP)-coupled sheep anti-mouse IgG (1:2000, 1h, room temperature) (GE Healthcare, Waukesha, WI, USA). Total IgG antibodies were detected with directly-labeled HRP anti-human IgG (1:5000, 1h, room temperature) (GE Healthcare). The color reaction was induced with 2,2′-azino-bis(3-ethyl-benzothiazoline-6-sulfonicacid)di-ammoniumsalt and optical density (*OD*_405nm_- *OD*_490nm_) was measured on a Spectra-Max-spectrophotometer (Molecular Devices, Sunnyvale, CA, USA).

Plate-to-plate normalization was done by testing control sera with established antibody levels for the antigens on each plate. Unspecific binding of detection antibodies to coated antigens was excluded by performing buffer controls (i.e., omission of sera). All determinations were carried out as duplicates with less than 5% variation and results are shown as normalized means of the raw *OD* data after subtraction of the reactivity to human serum albumin (HSA). As cut-off value (c.o.) for a positive reaction to a given antigen/peptide an *OD* value greater than the average *OD*+3SDs determined for ten HIV-uninfected subjects was defined.

Recombinant HIV-1 clade C proteins/peptides were printed on glass slides based on the Immuno Solid-phase Allergen Chip technology (Phadia GmbH, Vienna, Austria) to assemble micro-arrays for additional antibody measurements [29] (D. Gallerano, R. Valenta, unpublished).

### Structural modeling, sequence alignments and glycosylation predictions

The structure of South African HIV-1 clade C gp120 was generated with SWISS-MODEL [30]. As input we used the amino acid sequence of isolate ZA.04.04ZASK146 and a 3D model created with PyMOL (Version 1.3 Schrödinger, LLC) and COOT [31], in which the V3 loop of the gp120 structure PDB:2B4C was combined with the core, N- and C-termini of the gp120 structure PDB:3JWD. The V1/V2 domain (AA P123 to T191) was predicted to consist of mainly β-sheets by the program PSIpred [32]. Antibody-reactive peptides and CD4-binding sites [33] were marked using PyMOL. Multiple sequence alignments of peptides 120/15 and 120/24 from South African HIV-1 clade C with corresponding regions of HIV-1 reference strains were generated with Gene Doc (http://www.psc.edu/biomed/genedoc) and sequence identities were calculated with ClustalW2 (http://www.ebi.ac.uk/Tools/msa/clustalw2/). Sequences were numbered according to the international HXB2-numbering-scheme (http://www.hiv.lanl.gov). N-linked glycosylation sites were predicted for gp120 and gp41 proteins with NetNGlyc1.0 (http://www.cbs.dtu.dk/services/NetNGlyc/).

### Statistics

IBM SPSS Statistics 20.0 (Armonk, NY, USA) was used to analyze normalized data and to generate graphs. The antibody levels towards the single proteins and peptides were compared between European and African subjects by Mann Whitney *U* tests and the frequency of positive reactivity was compared by binomial tests (GraphPad Prism).

## Results

### HIV-infected patients from Africa and Europe show almost identical antibody responses towards gp120 peptide epitopes from HIV-1 clade C

**Fig 1:**
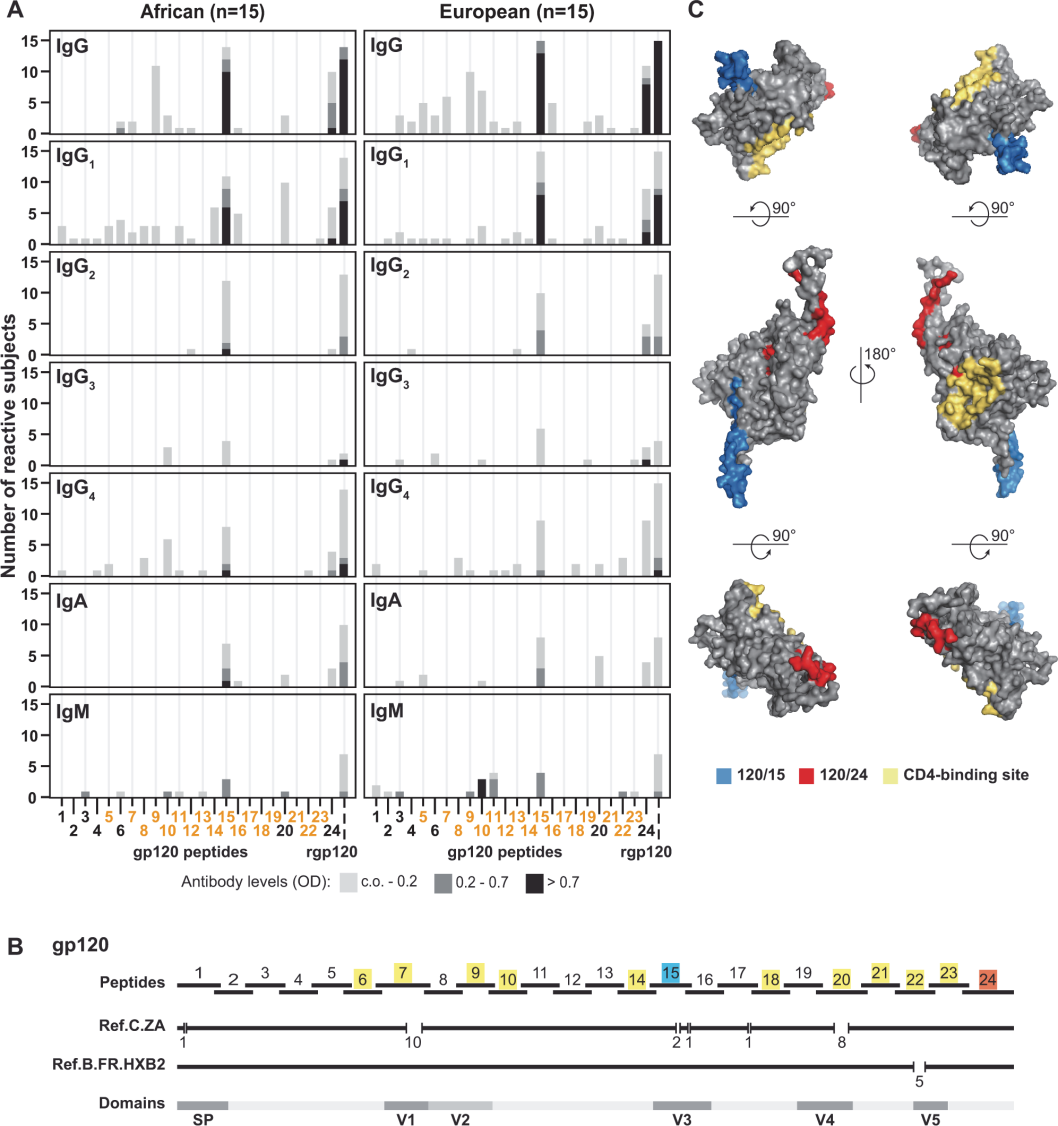
Antibody responses to HIV-1 clade C gp120 and gp120-derived peptides. (A) Frequencies and intensities (y-axes: number of reactive sera; *OD* levels color-coded) of IgG, IgG subclass, IgA and IgM responses of African (left) and European (right) patients to recombinant gp120 (rgp120) and 24 overlapping gp120 peptides (x-axes: peptides 1–24, peptides with predicted N-linked glycosylation sites: orange). (B) Position of overlapping gp120 peptides in gp120 of the HIV-1 clade C South African strain (Ref.C.ZA) and of the reference strain HXB2 (Ref.B.FR.HXB2). Gaps (numbers of missing amino acids are displayed) in gp120 from clade C and B required for optimal sequence alignment are indicated. Relevant protein domains described for gp120 clade B are indicated (SP, signal peptide; V1-V5, variable regions 1–5). Major IgG-reactive peptides 120/15 and 120/24 are indicated in blue and red, respectively and peptides containing amino acids involved in CD4 binding are shown in yellow. (C) Surface representation of the structural model of HIV-1 clade C gp120. Major antibody-reactive gp120 peptides (120/15: blue; 120/24: red) and amino acids involved in CD4 binding (yellow) are highlighted on different views of the model.

For a detailed epitope mapping of gp120 we tested sera from HIV-infected patients from Zimbabwe, where clade C is the predominant HIV-1 subtype [18], and from Europe where clade B is most common [17], for antibody reactivity with recombinant gp120 (rgp120) and 24 gp120-derived overlapping peptides (S1 Table). These peptides covered the complete amino acid sequence of gp120 derived from the South African HIV-1 subtype C reference strain. IgG, IgA, IgM and IgG_1–4_ subclass reactivities were measured (Fig. 1A, B).

Interestingly, IgG, IgG subclass, IgA and IgM response profiles towards the peptides were almost identical in the African (Fig. 1A, left panel) and in the European (Fig. 1A, right panel) HIV-infected patients. For gp120 the recognition pattern of the peptides was similar for all antibody classes and IgG subclasses. The analysis of antibody levels and frequencies of recognition showed that IgG and in particular IgG_1_ responses dominated in both populations but we found also IgG_2_, IgG_3_, IgG_4_, IgA and IgM responses against gp120 peptides in particular towards the most frequently recognized peptide 120/15 (Fig. 1A).

For IgG and IgG_1_, peptides 120/15 and 120/24 were the major antibody-reactive peptides in terms of frequency and intensity of recognition for the African and European patients (Fig. 1A). These peptides were recognized almost at the same frequency and intensity as the fully glycosylated rgp120. The overview of gp120 (Fig. 1B) shows that peptide 120/15 resides in the V3 domain, which contains a binding site for the co-receptor CCR5/CXCR4 on CD4 cells [34, 35] and that peptide 120/24 is located at the very C-terminal end of gp120.

S1 Fig contains a sequence alignment of peptides 120/15 and 120/24 with the corresponding gp120-derived regions from various HIV-1 reference strains from different continents (S3 Table). A high degree of sequence variation of the peptide 120/15-corresponding regions was found among the strains, ranging from 88 to 52% sequence identity. Depending on the strain, the peptide 120/15-defined region contains none, one or two predicted N-linked glycosylation sites (S1 Fig, left). The amino acid sequence of peptide 120/24 is more conserved among the strains than that of peptide 120/15 (i.e., >77% sequence identity) and does not include predicted N-linked glycosylation sites (S1 Fig, right). Peptide 120/9, which contains a cysteine residue involved in CD4 binding [33] (Fig. 1B, S1 Table) and which overlaps with the V2 domain (Fig. 1B), was also frequently recognized (i.e., by more than 70% of the patients), but the IgG-levels against peptide 120/9 were very low (Fig. 1A). 120/9-specific IgG_1_ was found only rarely, and we could not detect relevant IgG_2–4_, IgA or IgM reactivity specific for this peptide (Fig. 1A).

In order to visualize the position of the major antibody-reactive peptides on the three-dimensional structure of gp120, a structural model of the South African HIV-1 clade C gp120 was created (Fig. 1C). Peptides 120/15 and 120/24 comprise patches, which are in diametrical orientation on the structural model of the gp120 monomer (Fig. 1C). Highlighting the area representing the CD4-binding site of gp120 [33], we found that this area represented an independent surface patch between the regions defined by peptides 120/15 and 120/24 (Fig. 1C).

### African and European patients recognize highly similar epitopes on gp41 from HIV-1 clade C

**Fig 2:**
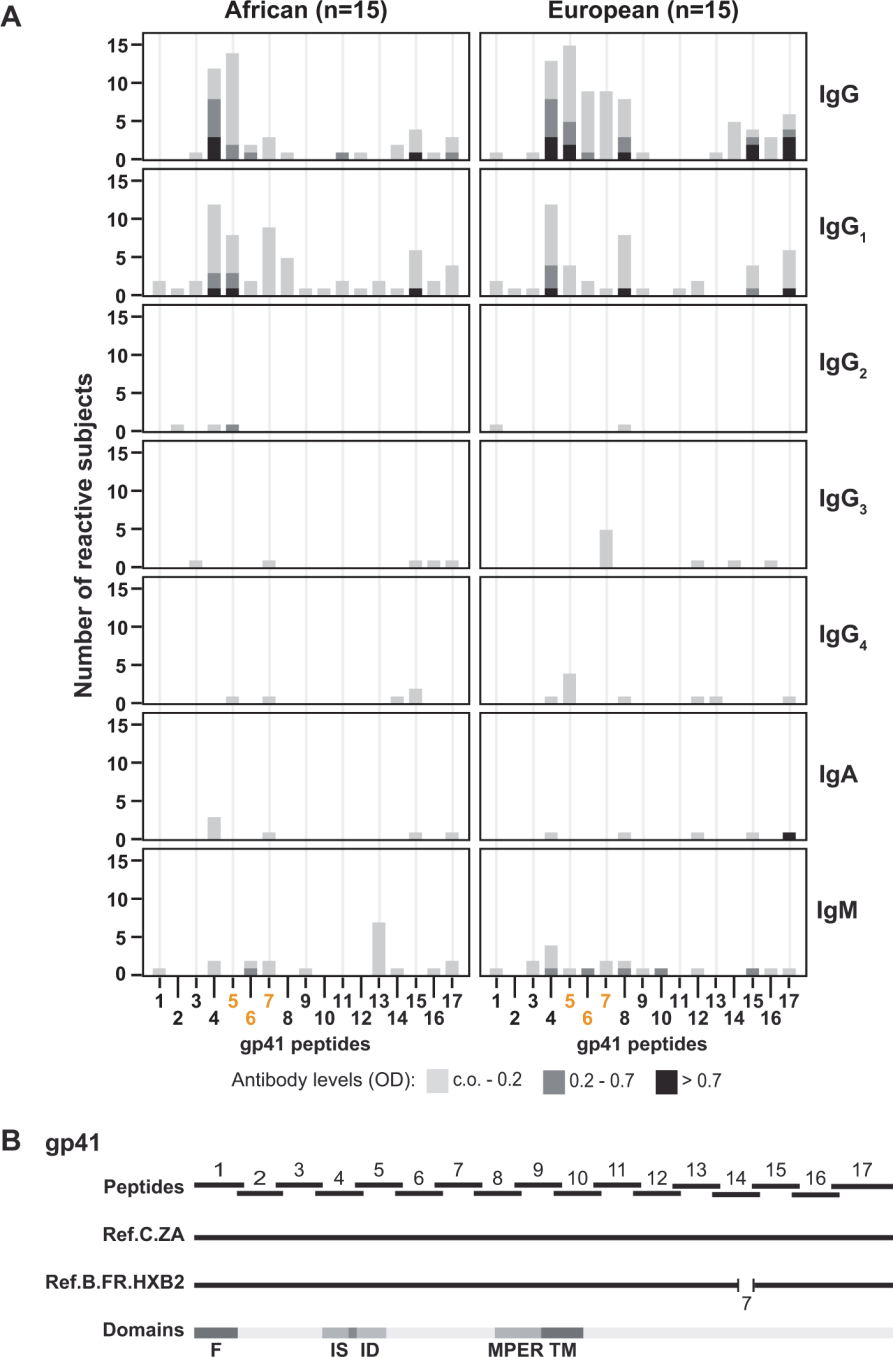
Antibody responses to gp160 and HIV-1 clade C gp41-derived peptides. (A) Frequencies and intensities (y-axes: number of reactive sera; *OD* levels color-coded) of IgG, IgG subclass, IgA and IgM responses of African (left) and European (right) patients to 17 overlapping gp41 peptides (x-axes: peptides 1–17, peptides with predicted N-linked glycosylation sites: orange). (B) Position of overlapping gp41 peptides in gp41 of the HIV-1 clade C South African strain (Ref.C.ZA) and of the reference strain HXB2 (Ref.B.FR.HXB2). A gap (number of missing amino acids is displayed) in gp41 from clade B, required for optimal sequence alignment, is indicated. Relevant protein domains/epitopes described for gp41 clade B are indicated (F, fusion peptide; ID, immunodominant region; IS, immunosuppressive region; MPER, membrane proximal external region; TM, transmembrane domain).

Next, we analyzed the IgG, IgA and IgM responses of the African and European patients to 17 overlapping peptides derived from HIV-1 clade C gp41 (Fig. 2A,B; S1 Table). Again we found that the African and European patients recognized similar peptides. The immune response was dominated by IgG and in particular by IgG_1_ antibodies (Fig. 2A). Both the frequency and intensity of recognition were lower towards gp41 peptides than to gp120-derived peptides. IgG and IgG_1_ antibodies were directed mainly to a region defined by peptides 41/4–8 but there was no relevant reactivity specific for these peptides in the IgG_2–4_ subclasses and regarding IgA and IgM (Fig. 2A). This was different from the antibody responses towards gp120 peptides, where we could detect reactivities against the major peptides epitopes in each of the four IgG subclasses, IgA and IgM (Fig. 1A). High IgG and IgG_1_ reactivity to peptides 41/04–05 is consistent with previous epitope mapping studies identifying the gp41 immunodominant domain [22, 36] (Fig. 2A,B). The other frequent antibody-reactive area was defined by peptides 41/14–17 and was located at the C-terminal portion of gp41, which is part of the “cytoplasmic domain” [37, 38] (Fig. 2A, B). Despite the overall similarity in the antibody reactivity profiles among African and European patients we observed also some differences in particular regarding peptides 41/6–8 and 41/16. Frequency of IgG recognition of 41/8-specific IgG (P = 0.0195) and levels (P = 0.0064) were significantly higher in European than in African patients and 41/6 as well as 41/16-specific IgG levels were significantly higher in European patients (P = 0.0009, P = 0.0281).

### African and European HIV-infected patients react primarily with structural and pol-derived proteins

**Fig 3:**
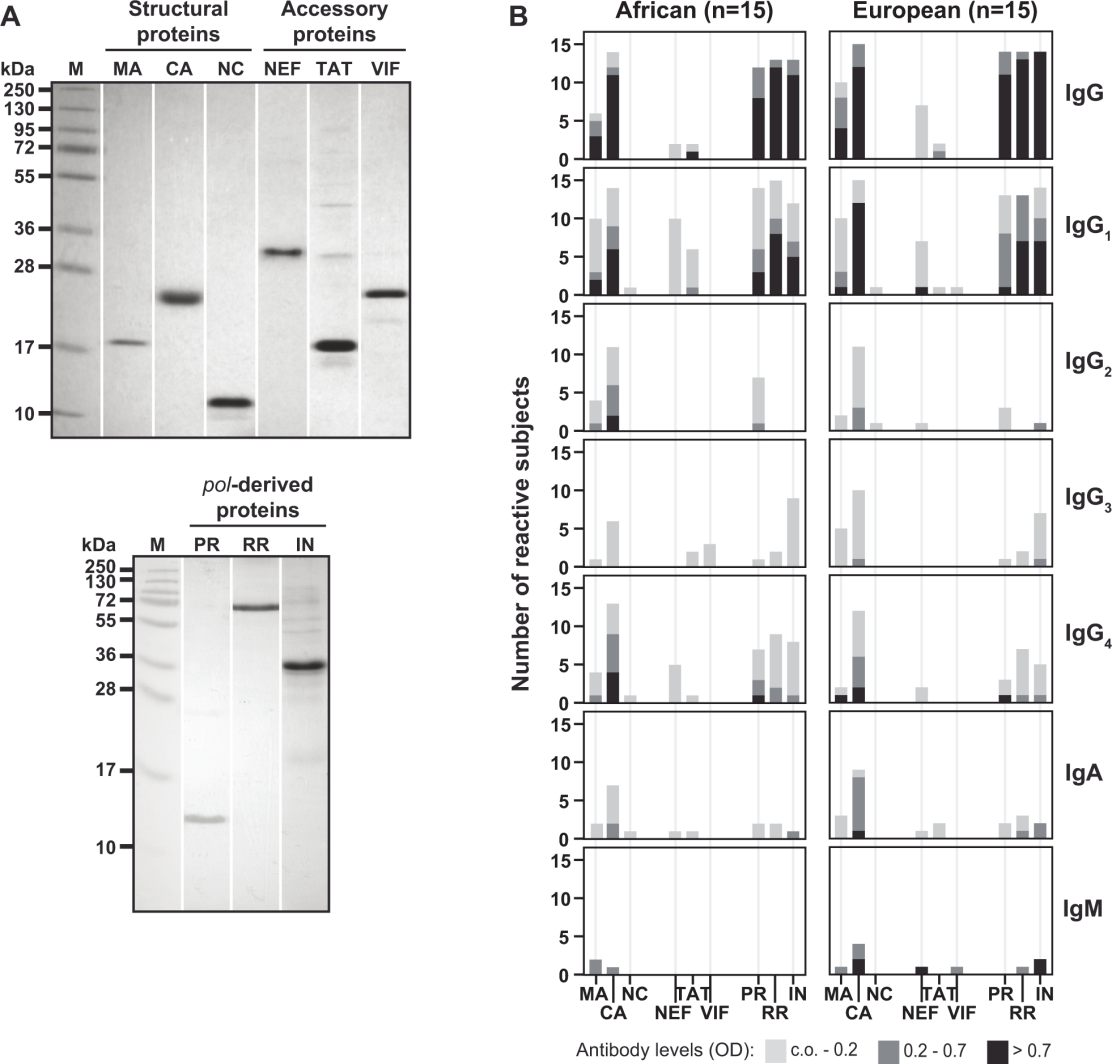
Purity and immunoreactivity of recombinant HIV-1 clade C structural, accessory and *pol*-derived proteins. (A) Coomassie-stained SDS-PAGE containing recombinant matrix (MA), capsid (CA), nucleocapsid (NC), NEF, TAT, VIF, protease (PR), reverse transcriptase+RNAseH (RR), integrase (IN) and molecular weight markers (M). Molecular weights (kDa) are indicated on the left. SDS-PAGE analysis of NEF was performed on a separate gel with identical molecular weight marker as for structural and accessory proteins. (B) Frequencies and intensities (y-axes: number of reactive sera; *OD* levels color-coded) of IgG, IgG subclass, IgA and IgM responses of African (left) and European (right) patients to structural, accessory and *pol*-derived proteins (x-axes).

In order to characterize the antibody response directed to HIV-1 clade C proteins other than the surface antigens, we expressed HIV-1 clade C structural, *pol*-derived and accessory proteins in *E*.*coli*. Each of the proteins was purified and, as determined by circular dichroism, was found to be folded (Fig. 3A; S4 Table). The folded proteins were then used to measure specific antibody levels in African (Fig. 3B, left panel) and European sera (Fig. 3B, right panel). Patients of both populations showed strong IgG and IgG1 responses with similar recognition profiles: The protease, reverse transcriptase+RNAseH, integrase, as well as the capsid and matrix proteins were the most frequently and strongly recognized antigens. IgG_2_, IgG_3_, IgG_4_, IgA and IgM responses against the latter antigens were also detected in African and European patients, albeit at lower frequencies and/or levels (Fig. 3B). In fact, we found moderate IgG_4_ reactivity towards *pol*-derived proteins in both populations, IgG_3_ towards integrase (in 8/15 African and 6/15 European) and IgG_2_ towards protease (in 7/15 African and 3/15 European subjects). IgM reactivity was detected only in few subjects. Antibody responses towards nucleocapsid and accessory proteins were found in only few patients and were of low intensity (Fig. 3B). When we compared specific IgG levels between the two populations we found significantly higher responses in European than African subjects for capsid (P = 0.0017), Nef (P = 0.0049) and integrase (P = 0.0328).

### IgG subclass recognition for gp120 peptides, structural and *pol*-derived proteins is indicative of a mixed Th1/Th2 immune response and accompanied by IgA and IgM responses

Regarding gp120, gp120 peptides, structural and *pol*-derived proteins, the antigen and epitope recognition profile was similar for all subclasses in the African and European patients (Fig. 1A, 2A, 3B). IgG_1_ responses were more intense and frequent than IgG_2_, IgG_3_ and IgG_4_ responses which can be explained by the fact that IgG_1_ represents the most abundant IgG subclass in serum. Frequencies and intensities of IgG_2_ and IgG_4_ responses were comparable, which is indicative of a mixed Th1/Th2 immune response [39, 40]. IgG and IgG subclass responses were accompanied by IgA and IgM responses. For gp41 peptides IgG and in particular IgG_1_ responses dominated and no relevant IgA and IgM responses were found.

### High sensitivity and specificity of diagnostic tests based on recombinant proteins and peptides assembling the clade C proteome

The comprehensive analysis of antibody responses showed that the array of HIV-1 clade C-derived antigens/peptides allowed the reliable detection of specific IgG antibodies in the HIV-infected patients from Africa and Europe (Fig. 1A, 2A, 3B and S2A Fig). The only negative serum was from a patient (i.e., patient #12) who was also negative in the InnoLIA Immunoblot-based test but had shown a severe CD4 cell reduction (S2 Table). In a follow up serum sample, obtained from patient #12 one year later, IgG against the peptides and rgp120 was found (as described in the next paragraph). No false-positive results were obtained when control-sera from uninfected individuals were tested. Peptides 120/15 and 41/5 were as good as rgp120 for IgG-based diagnosis of HIV-infected patients and 120/15-specific IgG-levels were comparable to rgp120-specific IgG-levels (S2A Fig). Using a panel of peptides 120/15, 120/24, rgp120, capsid and *pol*-derived proteins each of the infected patients was diagnosed by IgG determinations.

We also tested two asymptomatic highly exposed African individuals (i.e., patients #16, 17, S2 Table). Interestingly, these patients were negative in conventional analyses detecting p24-antigen and HIV-specific IgG/IgM and in Immunoblot-based assays. However, specific antibody responses could be identified by IgG and IgM measurements using the array of HIV-1 clade C antigens/peptides (S2B Fig).

### The IgG reactivity profile towards HIV-1 clade C proteome remains constant during disease and treatment in the majority of HIV-infected African patients

For 15 African patients for whom clinical documentation and sequential serum samples were available (i.e., patients #1–15, S2 Table) we monitored antibody specificities and levels during the course of infection and treatment. Patients were observed for periods ranging 3–44 months, with 2–4 serum samples taken per subject. For three patients (i.e., patients #2, 5, 10) sera were available before and after treatment, whereas the remaining patients were receiving anti-retroviral-therapy during the full monitoring period. Measurements of IgG antibodies against a panel of the most frequently recognized antigens/peptides revealed two forms of antibody responses over time.

**Fig 4:**
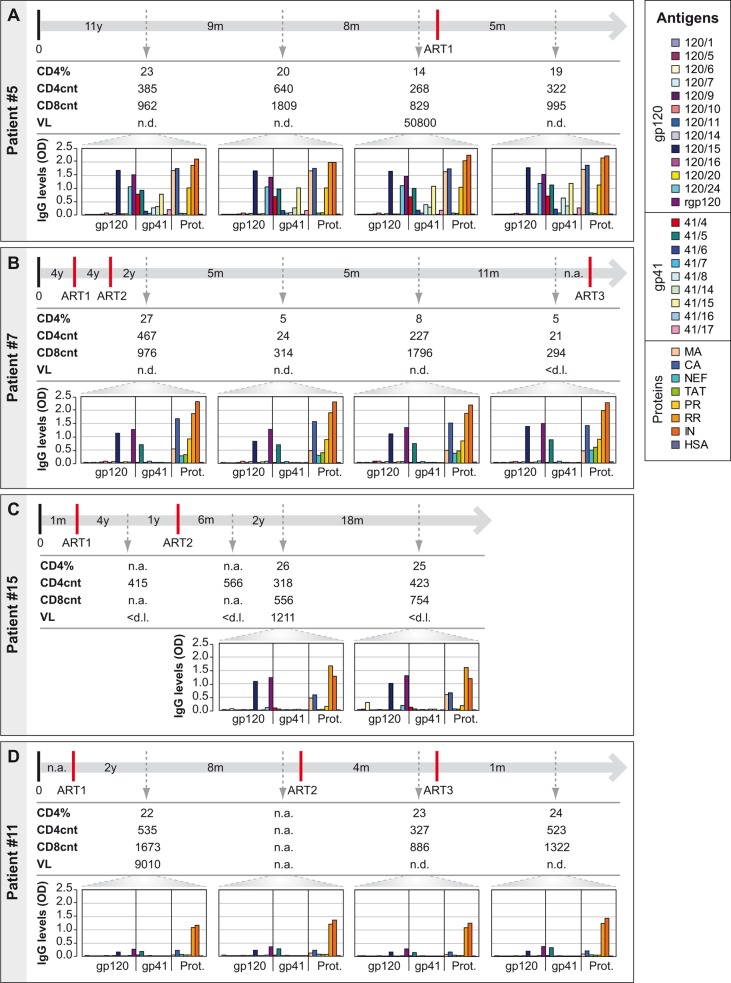
Monitoring of CD4+ and CD8+ T-cell counts, viral loads and IgG reactivity profiles to HIV-1 clade C-derived proteins and peptides during infection and treatment. Displayed are percentages of CD4+ T-cells, CD4+ and CD8+ T-cell counts, viral loads and levels of IgG antibodies to HIV-1 clade C-derived proteins and peptides (the box shows color codes of antigens and peptides) over time (top line) for patients with constant antibody reactivity profiles (A: patient #5; B: patient #7; C: patient #15; D: patient #11). Abbreviations: 0, 1^st^ positive test / 1^st^ visit; ART, antiretroviral therapy; ART1, ART2 and ART3 denote different ART regimens; y, years; m, months; VL, viral load; n.a., not available; n.d., not done; <d.l., below detection limit; *OD*, optical density; Prot., Proteins; MA, matrix; CA, capsid; NC, nucleocapsid; PR, protease; RR, reverse transcriptase+RNAseH; IN, integrase; HSA, human serum albumin.

In the majority of patients (i.e., 11/15 patients) antibody reactivity profiles remained constant in their specificity and overall intensity over the complete observation period. Fig. 4A-D show representative examples for patients monitored before and after treatment (Fig. 4A) and under anti-retroviral treatment (Fig. 4B-D). In these patients antibody specificities and levels remained constant even when viral load and CD4 counts changed. For example, no significant changes could be observed in the antibody response of patient #5 when CD4 counts declined between the second and third blood samples before therapy start, nor when CD4 counts rose 5 months post-treatment (Fig. 4A). Even more prominent was the example of patient #7 who experienced two severe drops of CD4 counts when the second and fourth blood samples were obtained but antibody profiles and levels remained largely unaltered (Fig. 4B). Furthermore, the specificities of the antibody profiles and the levels of the responses did not depend on viral loads found in blood samples. For example, in patient #15 between the third and fourth samples viral loads decreased from 1211 copies/ml to below the detection limit without showing an effect on antibody specificities or levels (Fig. 4C).

**Fig 5:**
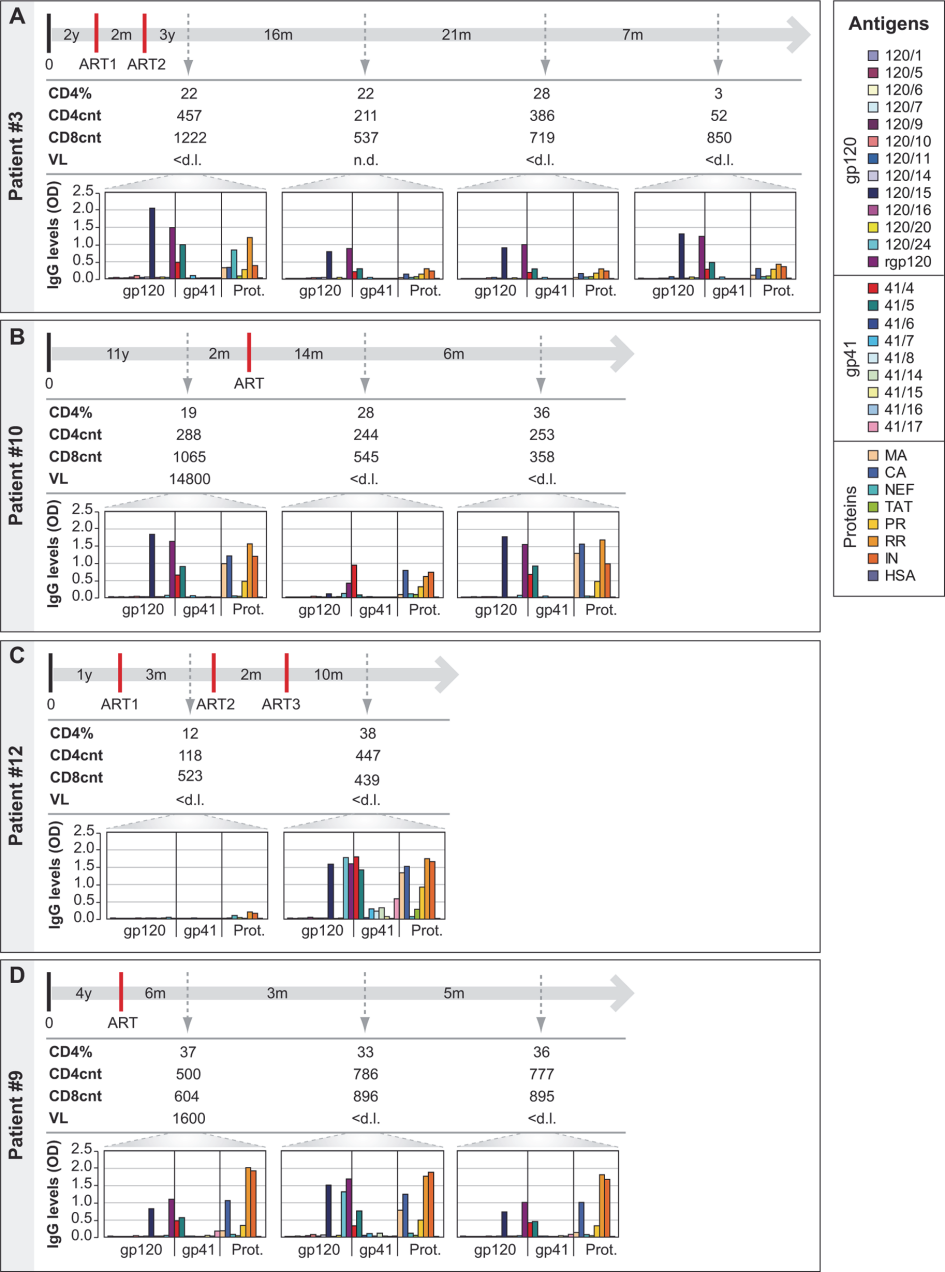
Monitoring of CD4+ and CD8+ T-cell counts, viral loads and IgG reactivity profiles to HIV-1 clade C-derived proteins and peptides during infection and treatment. Displayed are percentages of CD4+ T-cells, CD4+ and CD8+ T-cell counts, viral loads and levels of IgG antibodies to HIV-1 clade C-derived proteins and peptides (the box shows color codes of antigens and peptides) over time (top line) for patients with variable antibody reactivity profiles (A: patient #3; B: patient #10; C: patient #12; D: patient #9). Abbreviations: 0, 1^st^ positive test / 1^st^ visit; ART, antiretroviral therapy; ART1, ART2 and ART3 denote different ART regimens; y, years; m, months; VL, viral load; n.a., not available; n.d., not done; <d.l., below detection limit; *OD*, optical density; Prot., Proteins; MA, matrix; CA, capsid; NC, nucleocapsid; PR, protease; RR, reverse transcriptase+RNAseH; IN, integrase; HSA, human serum albumin.

In only four of the 15 patients we could observe changes of antibody specificities and levels (Fig. 5A-D). Patient #3 showed a reduction of IgG-levels towards each of the antigens/peptides. In this patient during the observation period viral loads were undetectable and CD4 counts varied (Fig. 5A). Patient #10 showed a decrease and then an increase of antibody levels towards peptides 120/15, 41/5, rgp120 and the matrix protein, while CD4 counts were unaltered and viral loads decreased below the detection limit (Fig. 5B). In patient #12 a strong increase of antibody levels was found in the second serum sample. CD4 counts increased and viral loads were below the detection limit during the observation period (Fig. 5C). In patient #9 we observed the appearance and disappearance of IgG reactivity towards peptide 120/24. CD4 counts were relatively constant and viral loads decreased (Fig. 5D). The profiles of antibody reactivities and the IgG-levels directed towards the individual components thus seemed not to be associated with CD4 counts or viral loads in the blood.

### Evidence for the persistence of IgG reactivity towards clade C proteins and peptides despite severe CD4 cell loss

In two patients (i.e., patients #7 and #3) we observed strong declines of CD4 cells (Fig. 4B, 5A). Interestingly, patient #7 showed a severe drop of CD4 counts in the second and fourth blood samples whereas CD4 counts had increased in the third blood sample, but the specificities and levels of antibodies remained unchanged (Fig. 4B). Likewise, patient #3 showed a strong decrease of CD4 counts from 386 to 52 when the third and fourth blood samples were analyzed, but again IgG specificities and levels did not change in a relevant manner between these time-points (Fig. 5A). These data indicate that HIV-specific antibody responses persist even after severe CD4 cell losses.

## Discussion

In this study we assembled the proteome of HIV-1 clade C by expressing, synthesizing and purifying a panel of structural, accessory and *pol*-derived proteins as well as overlapping peptides covering the virus envelope proteins gp120 and gp41. We then used this panel of proteins/peptides to compare epitope specificity, type and magnitude of antibody responses in a population of HIV-infected individuals from Sub-Saharan Africa (i.e., Zimbabwe) where clade C predominates and in another population from Europe, where clade B is prevalent. Despite the high degree of genetic variation among HIV-1 clades occurring in Africa and Europe [17], we found that patients from both areas showed almost identical antibody reactivities to clade C antigens/peptide epitopes. This finding was unexpected because the European patients’ group exposed to clade B was included only for comparison. Also magnitude and type of antibody responses (i.e., antibody class, subclass) were highly similar in both groups of patients. A limitation of the study was that, genetic information of the strains by which the patients were infected was not available to us. However, detailed prevalence studies among HIV-infected patients from Zimbabwe demonstrated the predominance of HIV-1 subtype C infections in this country [18].

The antibody responses were directed towards a few peptide epitopes of gp120 and gp41 which have been also described in studies analyzing epitopes of gp120 and gp41 from other HIV strains, suggesting that they indeed are the major targets of the polyclonal antibody responses of HIV-infected patients [20–24]. Peptides 120/15 and 120/24 were recognized almost at the same frequency and intensity as the fully glycosylated rgp120 by the patients polyclonal antibody response. Further experiments testing antibody binding to rgp120 upon after removal of peptide-specific antibodies may allow determining the proportion of gp120-specific antibody responses which are directed against these peptide epitopes. The strong recognition of clade C-derived peptide 120/15 also by European patients was surprising considering the high sequence variation of the peptide 120/15-corresponding regions among clades occurring in Europe and Africa. However, cross-reactivity towards the V3 region, in which peptide 120/15 resides, was underlined also in a recent study analyzing the antibody responses in sera from HIV vaccine trials and from an infected control group with overlapping peptides [25]. The second highly reactive gp120-derived peptide 120/24 showed lower variation of sequence homology among clades and the more weakly recognized peptide 120/9 overlaps with the V2 domain that has been reported as an epitope frequently recognized by individuals vaccinated with a recently developed gp120-based vaccine [41]. The gp41-derived peptides that were most frequently and strongly recognized were 41/4–8 and 41/14–17. These results are consistent with early reports characterizing the immunodominant and very C-terminal epitopes of gp41 [22, 36], overlapping with peptides 41/4, 5 and 17. Our results show that for HIV-1 clade C peptides flanking these regions are also frequently recognized and that differences can be observed in the European and African subjects. Furthermore, in samples of HIV-infected patients there was a pronounced antibody response towards structural and *pol*-derived but not to accessory proteins. These viral components may become immunogenic upon destruction of infected cells, through secretion from or by insertion into the membrane of infected cells. Structural and *pol*-derived proteins occur in far larger copy numbers than accessory proteins [42–45], which may explain why they are more immunogenic than accessory proteins.

An interesting difference regarding the IgG subclass, IgA and IgM responses was found regarding the different peptides and proteins. In fact, the major peptide epitopes 120/15 of gp120 was recognized by all antibody classes and IgG subclasses (i.e., IgG_1_>IgG_2_ = IgG_4_>IgG_3_, IgA>IgM). A broad IgG subclass response was also found for the C-terminal gp120 epitope and the non-surface proteins, protease, reverse transcriptase+RNAseH, integrase, as well as the capsid and matrix proteins. The IgG subclass for these peptides and antigens was dominated by IgG_1_, IgG_2_ and IgG_4_, which is indicative of a mixed Th1/Th2 response. These findings are in agreement with previous studies showing no evidence for a shift from a Th1 to a Th2 cytokine profile during HIV infection [46–48]. In fact, Th2 cells produce IL4 that induces a switch to IgG_4_ and IgE synthesis in B cells [49–51] whereas Th1 cells produce IFN gamma that has been shown to up-regulate IgG_2_ synthesis in human cells [51–52]. Both IL4 and IFNy inhibit the production of their phenotypic counterpart, i.e. of subclasses dependent on Th1 and Th2 cytokines respectively [39]. The subclass distribution observed in our study, in particular the presence of both IgG_2_ and IgG_4_ responses, may therefore be considered as being indicative of a mixed Th1/Th2 response.

gp41-derived-peptides were mainly recognized by IgG_1_ but not by the other IgG subclasses, IgA or IgM. One possibility for the low IgG_2–4_ subclass responses may be that total IgG levels towards the gp41 peptides and accessory proteins were also low. However, if indeed the IgG_2–4_ responses are low as compared to IgG_1_ this could be of relevance considering the different effector functions of the antibody classes and subclasses such as complement activation, ADCC, immune complex formation and defense at mucosal sites. We therefore suggest to further develop multiplex assays based on large panels of HIV proteins/peptides for measuring the full spectrum of isotypes and IgG subclasses similar as have been developed for chip-based allergen tests based on microarrayed antigens/peptides for larger cohort studies [53]. Results obtained with clade C-based panel recombinant proteins/peptides regarding diagnosis were encouraging because they showed that the tested panel was even more sensitive in diagnosing HIV-1 infections than commercial diagnostic HIV-tests. A limitation of the ELISA analysis of IgG, IgA, IgM and IgG subclass- reactivities towards a large panel of antigens/peptides is that quite large volumes of serum and time-consuming pipetting are required. We therefore started to employ micro-array technology as has been developed for diagnosis of allergy and autoimmune diseases for the comprehensive analysis of sera from HIV–infected patients [29, 53, 54]. Our first data indeed indicate that such microarrays can be used for the simultaneous measurement of antibody reactivities towards the full panel of antigens and peptides with only few microliters of serum requiring test durations of less than 3 hours (D. Gallerano, R. Valenta, unpublished). Another relevant observation in our study was that, although changes in antibody profiles were observed in rare cases, in the majority of HIV-infected patients epitope specificities remained constant over extensive periods with or without anti-retroviral therapy and even persisted despite severe T-cell loss. The latter is very interesting because it indicates that secondary HIV-specific IgG responses are not entirely dependent on CD4 help. Similar findings have been made for secondary IgE responses in experimental animal models for IgE-mediated allergy [55] and in allergic patients suffering from AIDS [56]. This result could be potentially important for vaccination strategies for HIV because it has been shown in other models that antibody responses can be boosted without T cell help [57].

Our study thus enriches the large body of knowledge regarding HIV-specific antibody responses with new information regarding epitope and antigen-specificity of isotype and subclass responses in HIV-infected patients from two continents who are exposed to different repertoires of strains. It also indicates that diagnostic tools based on comprehensive panels of recombinant HIV proteins and peptides are potentially useful also for studying HIV-specific immune responses in detail.

## Supporting Information

S1 Fig Multiple sequence alignments of antibody-reactive gp120 peptides. Alignments of the amino acid sequences of peptides 120/15 and 120/24 of HIV-1 clade C South African strain (Ref.C.ZA) with corresponding peptides from HIV-1 reference strains (S3 Table). Sequences are numbered accordingly to the HXB2 numbering scheme (top). Isolates from Africa and Europe are indicated in dark and light grey, respectively. Percentages of sequence identity with the Ref. C. ZA peptides are indicated on the right margin. Dots are conserved amino acids, dashes are gaps, predicted N-linked glycosylation sites are in orange and the “tip of the V3 domain” (V3tip) is boxed.
(TIF)

S2 Fig (A) IgG levels specific for rgp120, 120/15 and 41/5 in symptomatic HIV-infected patients and uninfected individuals. Shown are IgG levels expressed as optical density (OD, shaded in grey scale) detected against recombinant gp120 (rgp120) and peptides 120/15 and 41/5 in HIV-infected patients from Africa and Europe (two upper panels). Results obtained for HIV-uninfected subjects and controls with omission of sera (Buffer) are displayed in the two lower panels. C.o., cut-off; BC, buffer control. (B) IgG, IgG subclass, IgA and IgM reactivity profiles to gp120-derived proteins and peptides of two asymptomatic individuals with negative results in conventional HIV diagnostic tests. Shown are IgG, IgG1–4, IgA, and IgM antibody levels for positive antigens (rgp120; MA, matrix; NEF; TAT; PR, protease; RR, reverse transcriptase+RNAseH; IN, integrase) and peptides. Optical density (OD) levels are shown in different grey scales. C.o.: Cut-off. Negative test results obtained with the InnoLIA IgG immunoblot for HIV-1 antigens gp120, gp41, integrase (IN), capsid (CA), matrix (MA) and HIV-2 antigens gp105, gp36 are shown on the right margin.
(TIF)

S1 Table HIV-1 clade C gp120- and gp41-derived peptides. (DOC)

S2 Table Demographic, clinical and laboratory data of African subjects. (DOC)

S3 Table HIV-1 reference strains shown in S1 Fig.
(DOC)

S4 Table Biochemical features of recombinant HIV-1 clade C proteins. (DOC)
