# Supplementary figures and images for: Comparison of the Specificities of IgG, IgG-Subclass, IgA and IgM Reactivities in African and European HIV-Infected Individuals with an HIV-1 Clade C Proteome-Based Array
Source: PLoS One. 2015 Feb 6;10(2):e0117204. doi: 10.1371/journal.pone.0117204 (PMC4319756; doi:10.1371/journal.pone.0117204)

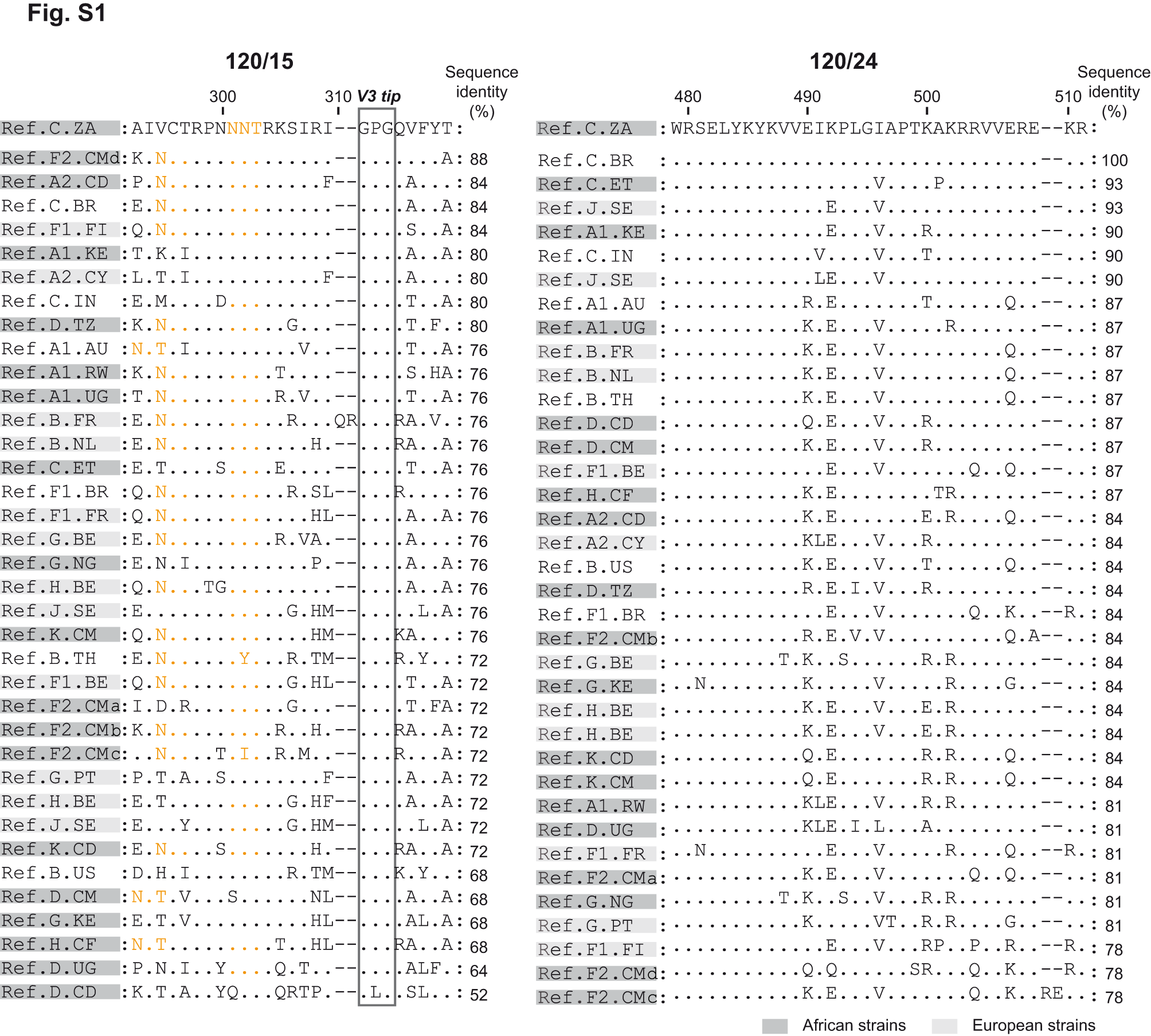

Supplement: S1 Fig — Alignments of the amino acid sequences of peptides 120/15 and 120/24 of HIV-1 clade C South African strain (Ref.C.ZA) with corresponding peptides from HIV-1 reference strains (S3 Table). Sequences are numbered accordingly to the HXB2 numbering scheme (top). Isolates from Africa and Europe are indicated in dark and light grey, respectively. Percentages of sequence identity with the Ref. C. ZA peptides are indicated on the right margin. Dots are conserved amino acids, dashes are gaps, predicted N-linked glycosylation sites are in orange and the “tip of the V3 domain” (V3tip) is boxed. (TIF) [file pone.0117204.s001.tif]

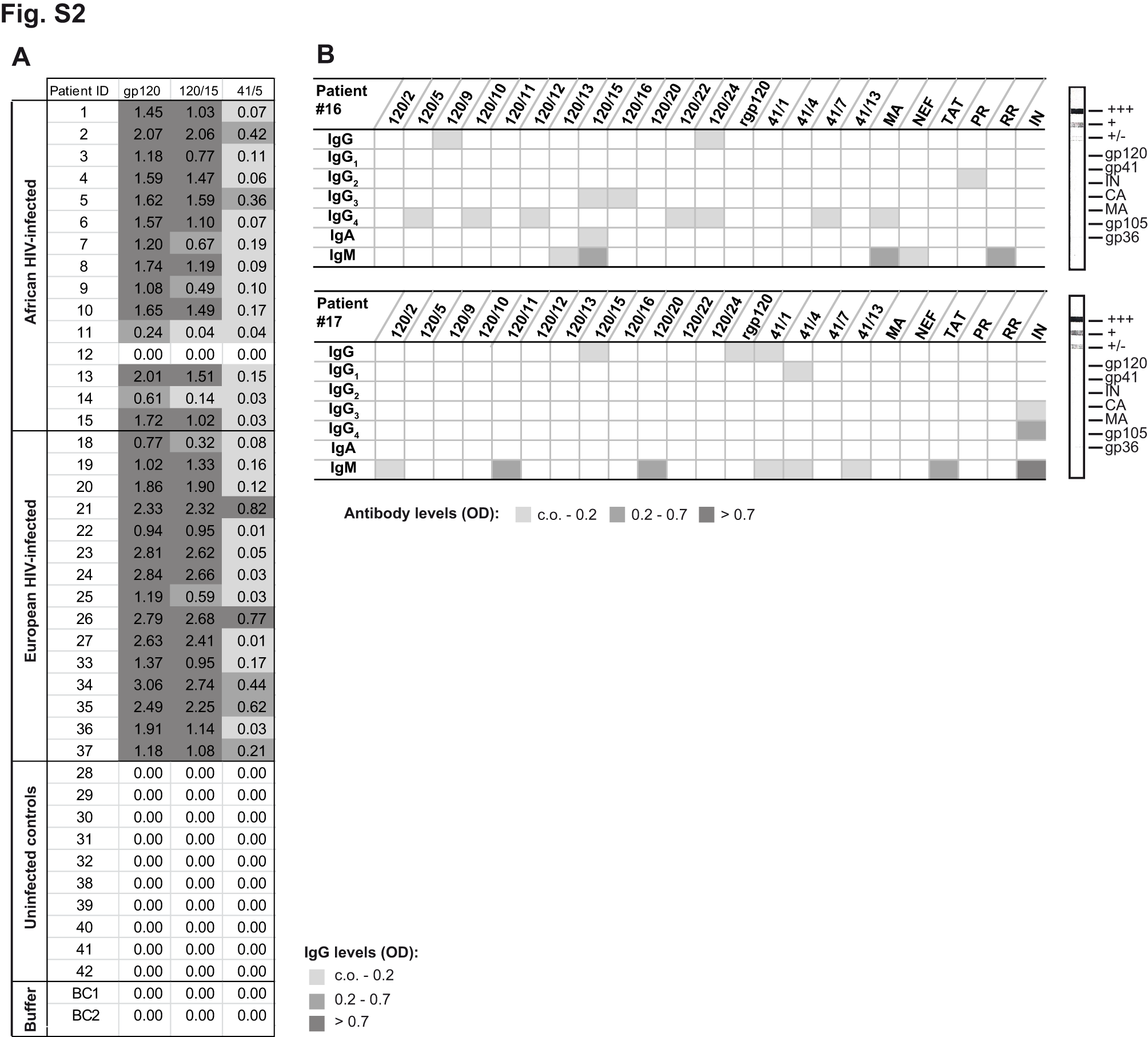

Supplement: S2 Fig — Shown are IgG levels expressed as optical density (OD, shaded in grey scale) detected against recombinant gp120 (rgp120) and peptides 120/15 and 41/5 in HIV-infected patients from Africa and Europe (two upper panels). Results obtained for HIV-uninfected subjects and controls with omission of sera (Buffer) are displayed in the two lower panels. C.o., cut-off; BC, buffer control. (B) IgG, IgG subclass, IgA and IgM reactivity profiles to gp120-derived proteins and peptides of two asymptomatic individuals with negative results in conventional HIV diagnostic tests. Shown are IgG, IgG1–4, IgA, and IgM antibody levels for positive antigens (rgp120; MA, matrix; NEF; TAT; PR, protease; RR, reverse transcriptase+RNAseH; IN, integrase) and peptides. Optical density (OD) levels are shown in different grey scales. C.o.: Cut-off. Negative test results obtained with the InnoLIA IgG immunoblot for HIV-1 antigens gp120, gp41, integrase (IN), capsid (CA), matrix (MA) and HIV-2 antigens gp105, gp36 are shown on the right margin. (TIF) [file pone.0117204.s002.tif]
