# Supplementary material for: Comparison of the Specificities of IgG, IgG-Subclass, IgA and IgM Reactivities in African and European HIV-Infected Individuals with an HIV-1 Clade C Proteome-Based Array
Source: PLoS One. 2015 Feb 6;10(2):e0117204. doi: 10.1371/journal.pone.0117204 (PMC4319756; doi:10.1371/journal.pone.0117204)
Supplement: S1 Table — (DOC) [file pone.0117204.s003.doc]

**Table S1. HIV**-1 clade C gp120- and gp41-derived peptides

| **Peptides** | **Position^A^** | **Amino acid sequence^B^** |
| --- | --- | --- |
| **120/1** | 2-28 | RVRGILRNWPQWWIWGILGFWMIII |
| **120/2** | 24b-47 | WMIIICRGEENSWVTVYYGVPVWTE |
| **120/3** | 43-67 | PVWTEAKTTLFCASDAKAYEKEVHN |
| **120/4** | 63-87 | KEVHNVWATHACVPTDPSPQELVLE |
| **120/5** | 83-107 | ELVLENVTESFNMWENDMVDQMHED |
| **120/6** | 103-127 | QMHEDIIGLWDESLKPCVKLT**PLCV** |
| **120/7** | 123-157 | T**PLCV**TLNCNTTSHNNSSPSPMTNC |
| **120/8** | 153-177 | PMTNCSFNATTELRDKTQKVNALFY |
| **120/9** | 173-197 | NALFYRSDIVPLEKNSSEYILIN**C**N |
| **120/10** | 193-217 | LIN**C**N**T**STITQACPKVSFDPIPIHY |
| **120/11** | 213-237 | IPIHYCAPAGYAILKCNNKTFNGTG |
| **120/12** | 233-257 | FNGTGPCSNVSTVQCTHGIKPVVST |
| **120/13** | 253-277 | PVVSTQLLLNGSLAEGEIIIRSENL |
| **120/14** | 273-297 | RSENLT**DNAKT**IIVHLNKSVAIVCT |
| **120/15** | 293-320 | AIVCTRPNNNTRKSIRIGPGQVFYT |
| **120/16** | 315-339 | QVFYTNEIIGNIRQAHCNISRELWN |
| **120/17** | 334-360 | RELWNNTLEQVKKKLKEHFQNKTIE |
| **120/18** | 356-380 | NKTIEFQPP**AGGD**L**E**VTTHSFNCRG |
| **120/19** | 376-400 | FNCRGEFFYCNTSNLFNITASNASD |
| **120/20** | 396-428 | SNASDANNNTITLPCKIKQII**NMW**Q |
| **120/21** | 424-448 | I**NMW**QE**VG**RAMYAPPIAGNITCNSS |
| **120/22** | 444-465e | TCNSSITGLLL**TRDGGN**NNDTGNNN |
| **120/23** | 465a-483 | TGNNNDTEIF**R**P**GGG**N**M**KDNWRSEL |
| **120/24** | 479-511 | WRSELYKYKVVEIKPLGIAPTKAKRRVVEREKR |
| **41/1** | 512-536 | AVGLGAVLLGFLGTAGSTMGAASIT |
| **41/2** | 532-556 | AASITLTVQARQLLSGIVQQQSNLL |
| **41/3** | 552-576 | QSNLLRAIEAQQHMLQLTVWGIKQL |
| **41/4** | 572-596 | GIKQLQARVLAIERYLKDQQLLGLW |
| **41/5** | 592-616 | LLGLWGCSGKLICTTAVHWNSSWSN |
| **41/6** | 612-636 | SSWSNKSQDYIWGNMTWMQWDREIN |
| **41/7** | 632-656 | DREINNYTDIIYTLLEESQSQQEKN |
| **41/8** | 652-676 | QQEKNEKDLLALDSWNNLWNWFSIT |
| **41/9** | 672-696 | WFSITKWLWYIKIFIMIVGGLIGLR |
| **41/10** | 692-716 | LIGLRIILGVLSIVKRVRQGYSPLS |
| **41/11** | 712-736 | YSPLSFQTLPPNPRGPDRLRGIEEE |
| **41/12** | 732-756 | GIEEEGGEQDKDRSIRLVSGFLALV |
| **41/13** | 752-776 | FLALVWEDLRSLCLFSYHRLRDFIL |
| **41/14** | 772-789 | RDFILIAGRAAELLGRSSLRGLQTG |
| **41/15** | 787e-809 | GLQTGWQALKYLGSLVQYWGLELKK |
| **41/16** | 805-829 | LELKKSAINLFDTTAIVVAEGTDRL |
| **41/17** | 825-856 | GTDRLIEGLQGIGRAIYNIPRRIRQGFEAALL |

^A^ Position of the peptides in gp120 and gp41, numbered according to the HXB2 numbering scheme. ^B^ Amino acids part of predicted N-linked glycosylation sites are underlined and gp120-derived amino acids involved in CD4 binding [33] and highlighted in Fig. 1C, are bold.
