# Supplementary material for: Comparison of the Specificities of IgG, IgG-Subclass, IgA and IgM Reactivities in African and European HIV-Infected Individuals with an HIV-1 Clade C Proteome-Based Array
Source: PLoS One. 2015 Feb 6;10(2):e0117204. doi: 10.1371/journal.pone.0117204 (PMC4319756; doi:10.1371/journal.pone.0117204)
Supplement: S2 Table — (DOC) [file pone.0117204.s004.doc]

**Table S2. Demographic**, clinical and laboratory data of African subjects

| **ID** | **Gender** | **Year**  **of birth** | **Year of**  **1^st^ +HIV test /**  **1^st^ visit** | **CD4 count**  **(*cells/ul*)**  **at 1^st^ +HIV test /**  **at 1^st^ visit** | **Time**  **without**  **ART** | **Year**  **of**  **ART**  **start** | **Observation**  **period:**  **Sampling**  **dates** | **Time**  **on ART** | **CD4 count**  ***cells/ul*** | **CD8 count**  ***cells/ul*** | **VL**  ***copies/ml*** |
| --- | --- | --- | --- | --- | --- | --- | --- | --- | --- | --- | --- |
| **1** | M | 1961 | n.a./2001 | n.a./114 | 4y | 2005 | 26.01.2009 | **~** 4y | 422 | 777 | < d.l. |
|  |  |  |  |  |  |  | 28.08.2009 | **~** 4y | 404 | 730 | < d.l. |
|  |  |  |  |  |  |  | 14.05.2010 | **~** 5y | 391 | 625 | < d.l. |
| **2** | M | 1950 | 2004/2004 | 668/668 | 6y | 2010 | 23.02.2010 |  | 346 | 1038 | 49500 |
|  |  |  |  |  |  |  | 03.06.2010 | **~** 4m | 577 | 1000 | n.d. |
| **3** | F | 1972 | n.a./2001 | n.a./171 | 2y | 2003 | 14.07.2006 | **~** 3y | 457 | 1222 | < d.l. |
|  |  |  |  |  |  |  | 16.04.2009 | **~** 6y | 211 | 537 | n.d. |
|  |  |  |  |  |  |  | 24.08.2009 | **~** 6y | 386 | 719 | < d.l. |
|  |  |  |  |  |  |  | 15.03.2010 | **~** 7y | 52 | 850 | < d.l. |
| **4** | M | 1974 | 1993/2001 | 1039/n.a. | 8y | 2001 | 09.04.2009 | **~** 8y | 876 | 584 | n.d. |
|  |  |  |  |  |  |  | 25.06.2010 | **~** 9y | 798 | 569 | < d.l. |
| **5** | M | 1949 | n.a./1997 | n.a./398 | 13y | 2010 | 02.10.2008 |  | 385 | 962 | n.d. |
|  |  |  |  |  |  |  | 28.07.2009 |  | 640 | 1809 | n.d. |
|  |  |  |  |  |  |  | 09.03.2010 |  | 268 | 829 | 50800 |
|  |  |  |  |  |  |  | 03.08.2010 | **~** 5m | 322 | 995 | n.d. |
| **6** | F | 1975 | n.a./2004 | n.a./32 | <1y | 2004 | 24.04.2009 | **~** 5y | 369 | 363 | n.d. |
|  |  |  |  |  |  |  | 11.09.2009 | **~** 5y | 777 | 1175 | n.d. |
|  |  |  |  |  |  |  | 05.08.2010 | **~** 6y | 500 | 237 | n.d. |
| **7** | M | 1971 | 1998/2002 | 168/n.a. | 4y | 2002 | 03.11.2008 | ~ 6y | 467 | 976 | n.d. |
|  |  |  |  |  |  |  | 06.04.2009 | ~ 7y | 24 | 314 | n.d. |
|  |  |  |  |  |  |  | 17.09.2009 | ~ 7y | 227 | 1796 | n.d. |
|  |  |  |  |  |  |  | 20.07.2010 | ~ 8y | 21 | 294 | n.d. |
| **8** | F | 1983 | n.a./2009 | n.a./136 | n.a. | 2009 | 27.05.2009 | ~ 1m | 472 | 674 | 75930 |
|  |  |  |  |  |  |  | 30.10.2009 | ~ 6m | 293 | 621 | n.d. |
|  |  |  |  |  |  |  | 15.04.2010 | ~ 1y | 261 | 875 | 20700^A^ |
|  |  |  |  |  |  |  | 01.02.2011 | ~ 2y | 741 | 960 | < d.l. |
| **9** | F | 1977 | n.a./2005 | n.a./684 | 4y | 2009 | 13.11.2009 | ~ 6m | 500 | 604 | 1600 |
|  |  |  |  |  |  |  | 05.02.2010 | ~ 9m | 786 | 896 | < d.l. |
|  |  |  |  |  |  |  | 05.07.2010 | ~ 1y | 777 | 895 | < d.l. |
| **10** | M | 1966 | 1998/1998 | 461/461 | 12y | 2010 | 30.11.2009 |  | 288 | 1065 | 14800 |
|  |  |  |  |  |  |  | 01.03.2011 | ~ 1y | 244 | 545 | < d.l. |
|  |  |  |  |  |  |  | 09.09.2011 | ~ 1.5y | 253 | 358 | < d.l. |
| **11** | M | 1968 | 2008/2008 | 210/210 | <1y | 2008 | 06.05.2010 | ~ 2y | 535 | 1673 | 9010 |
|  |  |  |  |  |  |  | 10.01.2011 | ~ 3y | n.a. | n.a. | n.d. |
|  |  |  |  |  |  |  | 06.05.2011 | ~ 3y | 327 | 886 | n.d. |
|  |  |  |  |  |  |  | 13.06.2011 | ~ 3y | 523 | 1322 | n.d. |
| **12** | M | 1961 | 2009/2010 | 250/n.a. | 1y | 2010 | 24.05.2010 | ~ 3m | 118 | 523 | < d.l. |
|  |  |  |  |  |  |  | 10.05.2011 | ~ 1y | 447 | 439 | < d.l. |
| **13** | M | 1953 | n.a./1998 | n.a./277 | <1y | 1998 | 06.04.2009 | ~ 11y | 206 | 192 | n.d. |
|  |  |  |  |  |  |  | 18.06.2010 | ~ 12y | 593 | 624 | 711 |
|  |  |  |  |  |  |  | 15.03.2011 | ~ 13y | 523 | 726 | n.d. |
| **14** | F | 1975 | 2003/2004 | 312/n.a. | 1y | 2004 | 14.01.2010 | ~ 6y | 526 | 1191 | n.d. |
|  |  |  |  |  |  |  | 24.06.2010 | ~ 6y | 395 | 887 | < d.l. |
|  |  |  |  |  |  |  | 23.02.2011 | ~ 7y | 776 | 1688 | n.d. |
| **15** | M | 1957 | 2001/1999 | 154/n.a. | <1y | 2001 | 23.03.2009 | ~ 8y | 318 | 556 | 1211 |
|  |  |  |  |  |  |  | 07.09.2010 | ~ 9y | 423 | 754 | < d.l. |
| **16** | F | 1958 | n.a./2010 | n.a./713 |  | Not on ART | 05.07.2010 |  | 713 | 321 | n.d. |
| **17** | M | 1970 | n.a./2009 | n.a./1365 |  | Not on ART | 21.12.2009 |  | 1365 | 622 | n.d. |

Abbreviations: F, female; M, male; y, years; m, months; VL, plasma viral load; n.a., not available; n.d., not done; < d.l., below detection limit. ^A^VL determined on a sample from June 2010.
