# Supplementary material for: Comparison of the Specificities of IgG, IgG-Subclass, IgA and IgM Reactivities in African and European HIV-Infected Individuals with an HIV-1 Clade C Proteome-Based Array
Source: PLoS One. 2015 Feb 6;10(2):e0117204. doi: 10.1371/journal.pone.0117204 (PMC4319756; doi:10.1371/journal.pone.0117204)
Supplement: S3 Table — (DOC) [file pone.0117204.s005.doc]

**Table S3. HIV-1 reference strains shown in Figure S1**

| **Abbreviation** | **Subtype** | **Sampling country** | **Sampling year** | **Accession number^A^** | **ID** |
| --- | --- | --- | --- | --- | --- |
| Ref.A1.AU | A1 | Australia | 2003 | DQ676872 | PS1044_Day0 |
| Ref.A1.KE | A1 | Kenya | 1994 | AF004885 | Q23_17 |
| Ref.A1.RW | A1 | Rwanda | 1992 | AB253421 | 92RW008 |
| Ref.A1.UG | A1 | Uganda | 1992 | AB253429 | 92UG037 |
| Ref.A2.CD | A2 | Dem. Rep. of Congo | 1997 | AF286238 | 97CDKTB48 |
| Ref.A2.CY | A2 | Cyprus | 1994 | AF286237 | 94CY017_41 |
| Ref.B.FR | B | France | 1983 | K03455 | HXB2_LAI_IIIB_BRU |
| Ref.B.NL | B | Netherlands | 2000 | AY423387 | 671_00T36 |
| Ref.B.TH | B | Thailand | 1990 | AY173951 | BK132 |
| Ref.B.US | B | United States | 1998 | AY331295 | 1058_11 |
| Ref.C.BR | C | Brazil | 1992 | U52953 | BR025_d |
| Ref.C.ET | C | Ethiopia | 1986 | U46016 | ETH2220 |
| Ref.C.IN | C | India | 1995 | AF067155 | 95IN21068 |
| Ref.C.ZA | C | South Africa | 2004 | AY772699 | SK164B1 |
| Ref.D.CD | D | Dem. Rep. of Congo | 1983 | K03454 | ELI |
| Ref.D.CM | D | Cameroon | 2001 | AY371157 | 01CM_4412HAL |
| Ref.D.TZ | D | Tanzania | 2001 | AY253311 | A280 |
| Ref.D.UG | D | Uganda | 1994 | U88824 | 94UG114 |
| Ref.F1.BE | F1 | Belgium | 1993 | AF077336 | VI850 |
| Ref.F1.BR | F1 | Brazil | 1993 | AF005494 | 93BR020_1 |
| Ref.F1.FI | F1 | Finland | 1993 | AF075703 | FIN9363 |
| Ref.F1.FR | F1 | France | 1996 | AJ249238 | MP411 |
| Ref.F2.CM a | F2 | Cameroon | 1995 | AJ249236 | MP255 |
| Ref.F2.CM.b | F2 | Cameroon | 1995 | AJ249237 | MP257 |
| Ref.F2.CM c | F2 | Cameroon | 1997 | AF377956 | CM53657 |
| Ref.F2.CM d | F2 | Cameroon | 2002 | AY371158 | 02CM_0016BBY |
| Ref.G.BE | G | Belgium | 1996 | AF084936 | DRCBL |
| Ref.G.KE | G | Kenya | 1993 | AF061641 | HH8793_12_1 |
| Ref.G.NG | G | Nigeria | 1992 | U88826 | 92NG083 |
| Ref.G.PT | G | Portugal | 2004 | AY612637 | PT2695 |
| Ref.H.BE a | H | Belgium | 1993 | AF190127 | VI991 |
| Ref.H.BE b | H | Belgium | 1993 | AF190128 | VI997 |
| Ref.H.CF | H | Central African Rep. | 1990 | AF005496 | 056 |
| Ref.J.SE a | J | Senegal | 1993 | AF082394 | SE7887 |
| Ref.J.SE b | J | Senegal | 1994 | AF082395 | SE7022 |
| Ref.K.CD | K | Dem. Rep. of Congo | 1997 | AJ249235 | EQTB11C |
| Ref.K.CM | K | Cameroon | 1996 | AJ249239 | MP535 |

^A^www.hiv.lanl.gov
