# Supplementary material for: Comparison of the Specificities of IgG, IgG-Subclass, IgA and IgM Reactivities in African and European HIV-Infected Individuals with an HIV-1 Clade C Proteome-Based Array
Source: PLoS One. 2015 Feb 6;10(2):e0117204. doi: 10.1371/journal.pone.0117204 (PMC4319756; doi:10.1371/journal.pone.0117204)
Supplement: S4 Table — (DOC) [file pone.0117204.s006.doc]

**Table S4. Biochemical features of recombinant HIV-1 clade C proteins**

| **Proteins** | **Predicted molecular weight**  ***(kDa)*** | **Calculated isoelectric point** | **Secondary structure**  **determined by**  **circular dichroism** | **Thermal stability**  **determined by**  **circular dichroism** |
| --- | --- | --- | --- | --- |
| **MA** | 15.5 | 9.1 | α-helical (63%), β-sheet (10%) | Tm = 65°C |
| **CA** | 26.5 | 6.6 | α-helical (57%), β-sheet (12%) | Tm = 66°C |
| **NC** | 7.2 | 10.2 | β-sheet (34%), α-helical (5%) | n.d. |
| **NEF** | 24.6 | 6.2 | β-sheet (27%), α-helical (14%) | n.d. |
| **TAT** | 12.2 | 9.0 | β-sheet (29%), α-helical (12%) | n.d. |
| **VIF** | 23.7 | 10.5 | β-sheet (32%), α-helical (12%) | Tm > 95°C |
| **PR** | 11.7 | 8.7 | β-sheet (42%), α-helical (6%) | Tm = 55°C |
| **RR** | 65.1 | 6.8 | β-sheet (28%), α-helical (17%) | Tm > 95°C |
| **IN** | 33.2 | 7.4 | β-sheet (25%), α-helical (19%) | n.d. |

Abbreviations: MA, matrix; CA, capsid; NC, nucleocapsid; PR, protease; RR, reverse transcriptase+RNAseH; IN, integrase; kDa, kilodalton; Tm, melting temperature; n.d., not done.
